# A global assessment of the gender gap in self-reported health with survey data from 59 countries

**DOI:** 10.1186/s12889-016-3352-y

**Published:** 2016-07-30

**Authors:** Ties Boerma, Ahmad Reza Hosseinpoor, Emese Verdes, Somnath Chatterji

**Affiliations:** Department of Information, Evidence and Research, World Health Organization, Geneva, Switzerland

**Keywords:** Self-reported health, Gender differences, Health surveys, Behavioural factors, Biological factors, Gender inequality, Chronic conditions

## Abstract

**Background:**

While surveys in high-income countries show that women generally have poorer self-reported health than men, much less is known about gender differences in other regions of the world. Such data can be used to examine the determinants of sex differences.

**Methods:**

We analysed data on respondents 18 years and over from the World Health Surveys 2002–04 in 59 countries, which included multiple measures of self-reported health, eight domains of functioning and presumptive diagnoses of chronic conditions. The age-standardized female excess fraction was computed for all indicators and analysed for five regional groups of countries. Multivariate regression models were used to examine the association between country gaps in self-reported health between the sexes with societal and other background characteristics.

**Results:**

Women reported significantly poorer health than men on all self-reported health indicators. The excess fraction was 15 % for the health score based on the eight domains, 28 % for “poor” or “very poor” self-rated health on the single question, and 26 % for “severe” or “extreme” on a single question on limitations. The excess female reporting of poorer health occurred at all ages, but was smaller at ages 60 and over. The female excess was observed in all regions, and was smallest in the European high-income countries. Women more frequently reported problems in specific health domains, with the excess fraction ranging from 25 % for vision to 35 % for mobility, pain and sleep, and with considerable variation between regions. Angina, arthritis and depression had female excess fractions of 33, 32 and 42 % respectively. Higher female prevalence of the presumptive diagnoses was observed in all regional country groups. The main factors affecting the size of the gender gap in self-reported health were the female-male gaps in the prevalence of chronic conditions, especially arthritis and depression and gender characteristics of the society.

**Conclusions:**

Large female-male differences in self-reported health and functioning, equivalent to a decade of growing older, consistently occurred in all regions of the world, irrespective of differences in mortality levels or societal factors. The multi-country study suggests that a mix of biological factors and societal gender inequalities are major contributing factors to gender gap in self-reported measures of health.

**Electronic supplementary material:**

The online version of this article (doi:10.1186/s12889-016-3352-y) contains supplementary material, which is available to authorized users.

## Background

Globally, women have an average life expectancy that is about 5 years longer than that men, as a result of lower mortality rates at all ages [1]. There is, however, also evidence from household surveys in mostly high-income countries indicating that women consistently report a poorer health status than men [2, 3]. As a result the gap between the sexes is smaller for measures that combine mortality and morbidity, such as healthy life expectancy (4 years at birth), than for life expectancy (5 years at birth) [4].

The existence of sex differences in mortality and morbidity has stimulated research into the extent to which gender roles are playing a role. The proposed explanations for the health-survival paradox are rooted in biological, social and psychological interpretations [5]. In this study the focus is on the gap in self-reported health of women and men, which has been attributed to a range of a combination of biological, socio- behavioural and psychological differences which may result in reporting differences [3]. Studies in mostly high-income countries suggest that physical health status differences as well as mental health issues are important underlying factors [3, 6–8]. Differences in sociobehavioural factors such as employment and educational differences between men and women have also shown to be associated with gender differences in self-reported health [9, 10], as well as risk behaviour patterns such as smoking [3]. Some have suggested that a reporting bias, associated with gender-associated psychosocial factors or with actual use of healthcare, contributes to a greater propensity to report poor health in surveys among women [11–13].

A global study of the existence and size of a gap in self-reported health between the sexes allows an assessment of the consistency of the patterns in widely divergent sociocultural and economic settings and provides insight into the extent to which societal characteristics may influence the size of the gaps. This study examines the gap with data on multiple indicators of self-reported health among respondents 18 years and over from 59 countries, using the World Health Survey 2002–2004. The World Health Survey included a range of questions on domains of self-reported health and symptoms of chronic conditions. An ecological analysis is conducted to assess which societal determinants may affect the self-reported health gap in in this group of highly variable socio-economic and cultural settings. This includes an assessment of the association with male–female differences in employment and education, as well as gender inequality.

## Methods

The World Health Survey (WHS) was conducted between 2002 and 2004 in 70 countries in all regions of the world and coordinated by the World Health Organization. The primary aim was to provide representative and comparable population data on the health status of adults 18 years and older. All countries conducted nationally representative surveys but in six the sample did not cover the whole country (China, Comoros, Congo, Cote d’Ivoire, India and Russia). The mean sample size was over 4000 respondents, ranging from about 1000 to over 20,000. Eleven countries were excluded from this analysis because of lack of data on sampling weights. The 59 countries included 143,363 male and 115,321 female respondents for the analysis. (For more details on the WHS, see www.who.int/healthinfo/survey/en/).

The surveys were classified into six country groups including sub-Saharan Africa (17 countries), high income countries in Europe (nine countries), middle income countries in Eastern Europe (nine countries), Latin America (six countries) and South Asia (six countries); the remaining 12 countries were categorised as other to allow concise presentation of the results. (see Additional file 1 for details). All regional and global statistics are weighted using post-sample stratification weights.

Three self-reported measures of health were used [14–16]. First, a health (state) score derived from 16 questions in eight health domains: mobility, vision, cognition, self-care, personal interrelationships, sleep, affect and pain. The full questionnaire module is provided in Additional file 2. The Rasch partial credit model of Item Response theory was used to construct a composite score [17]. The inverse of the score obtained from the model was transformed to a scale ranging from 0 (best) to 100 (worst) called the “poor-health” score in this paper.

The second measure was based on a single question of overall self-rated health: “How would you rate your health today?”, with responses on a five point Likert scale. The responses “bad” and “very bad” were considered to indicate poor self-rated health [18, 19]. Finally, respondents in 51 surveys were asked: “Overall in the last 30 days, how much difficulty did you have with work or household activities Respondents were considered to have significant limitations in day to day activities if the response was “severe” or “extreme”. Similar questions are included in widely used general population survey measures such as the EQ-5D-5 L and comparisons show that the two severe and extreme categories are equivalent to significant difficulties when only the three level version is used [20, 21].

In addition, symptom questions were asked to assess the prevalence of probable chronic conditions, based on recall of doctor’s diagnosis plus symptom questions among those with no recall of diagnosis. This included angina pectoris, based on the algorithm based on the Rose questionnaire [22, 23], asthma and arthritis, both based on algorithms derived from questionnaires used previously in general populations studies [24–26], and depression based on questions based on the depression module of the World Mental Health Survey version of the Composite International Diagnostic Interview [27]. For diabetes, self-reported diagnosis was used, as no satisfactory diagnostic algorithm based on symptoms is currently available.

The size and distribution of the gender gap by region was examined for the three measures of self-reported health, the eight domains health separately, and the survey-based diagnoses of chronic conditions using the (female) excess fraction (defined as female prevalence minus male prevalence, divided by female prevalence) [28].

In addition, the association between the gap in women and men’s self-rated health and selected socioeconomic, demographic and socio-behavioural factors was examined using aggregate country data on the following variables:

- Life expectancy gap (in years): based on WHO data for 2000 [1]). Mortality levels may have an association with the size of the gaps in self-reported health;
- Islamic countries: Islam is the majority religion (13 countries, see Additional file 1). Religion may be considered a proxy for societal and behavioural differences between men and women that affect self-reported health;
- Total Fertility Rate: number of children born to a woman at the end of her reproductive years, for the year 2000 [1]. Childbearing may have an impact on women’s health;
- Employment gap: difference in the proportion of men and women who reported themselves in formal employment in the WHS. Studies have shown the association between employment and health [9];
- Education gap: difference in mean number of years of education between men and women in the WHS. Education has a strong relationship with health in many settings;
- Gross Domestic Product (GDP) per capita: in international dollars, PPP adjusted, for the year 2000 [1];
- Gender inequality index: a composite measure produced by the United Nations Development Programme based on reproductive health (maternal mortality and adolescent birth rate), empowerment (based on secondary education levels among women compared to men and female occupation of parliamentary seats) and female labour force participation [29];
- Smoking gap: difference between male and female current daily smoking rates, data from the WHS 2002–04;
- Alcohol use gap: difference between male and female prevalence in current use of alcohol, data from the WHS 2002–04.

In all analyses age-standardized rates were used. Multivariate regression models with country aggregate data were used to examine the association between gender gaps in each of the three measures of health with demographic, socio-behavioural and biological factors. Because of collinearity between the different variables, we opted for a stepwise regression with a cut-off of 10 % to obtain insights into factors affecting the size of the gender gap. For the analysis of activity limitations we excluded Slovakia as it was a major outlier in the size of the gender gap affecting the results (a difference of 7 percentage points with women being worse as compared to the average pooled difference of about 3 percentage points). All analyses were weighted and conducted in Stata version 11.

Informed consent was obtained in all surveys. A standard consent form approved by the ethics review committee was read to the respondent in the respondent’s language. If the respondent was literate and gave consent to participate, the form was provided to the respondent to read and sign, and was countersigned by the interviewer. If the respondent was illiterate and gave consent to participate, the interviewer confirmed this consent by signing that the respondent had been read the form, understood the study, and agreed to participate. This procedure was approved by the institutional review boards in each study country. The anonymised datasets are in the public domain (http://apps.who.int/healthinfo/systems/surveydata/index.php/catalog/central) and this study is based on secondary analysis of these datasets and hence no additional ethical clearance was sought.

## Results

### Overall health

Table 1 presents the three self-reported health measures by sex, age and regional groups. For both men and women the prevalence of poor health increased with age. In general, respondents in the high-income countries in Europe and Latin America had lower self-reported prevalence of health problems than other regions.

**Table 1:** Self-reported health among men and women and female excess fraction, by age and country grouping: domain-based poor-health score, poor self-rated health and limitations in daily activities, WHS 2002–2004

	Poor health score			Poor self-rated health			Limitations in daily activities		
	Women (%)	Men (%)	Excess (%)^a^	Women (%)	Men (%)	Excess (%)^a^	Women (%)	Men (%)	Excess (%)^a^
Age (years)									
18–29	22.0	18.5	16	4.4	3.3	26	3.8	3.1	19
30–39	24.6	20.4	17	7.1	5.0	30	6.2	4.8	23
40–49	27.9	23.5	16	10.7	6.8	36	9.6	6.1	37
50–59	31.6	26.7	15	15.9	10.9	31	12.3	9.3	24
60–69	34.6	30.4	12	21.1	17.0	19	18.4	14.3	22
70–79	38.6	34.9	9	30.6	22.8	26	30.1	21.5	28
80+	42.2	38.6	9	34.2	26.9	21	43.0	35.1	18
Region									
Sub-Saharan Africa	28.8	24.9	14	13.8	10.1	27	11.3	9.5	16
Latin America	27.8	22.7	18	7.9	5.1	35	7.0	5.0	29
Europe, high-income	24.6	21.6	12	8.5	6.7	21	-	-	-
Eastern Europe	27.4	23.3	15	13.8	11.1	20	9.9	7.3	26
South Asia	27.2	22.9	16	10.1	6.6	35	9.8	6.7	32
Other	29.4	24.5	.17	12.4	8.0	35	13.4	8.1	40
Total	27.6	23.5	15	11.5	8.3	28	10.3	7.7	26

^a^Excess is the female excess fraction; for domain health score the inverse was used; All female-male differences are statistically significant at the 1 % level, as sample sizes of the pooled data sets are very large; All regional figures are age-standardized. -: only two countries in the region included the question

There was considerable variation between individual countries (see Additional file 3). Major outliers were Swaziland and Morocco which had much higher self-reported prevalence of health problems than all other countries. Association between the self-reported prevalence of poor health and life expectancy in countries was weak for all three measures and for both sexes (Pearson correlation coefficients ranging from -.16 to -.27). The three measures were closely correlated with each other. Among women, the correlation coefficients for the country aggregate scores were .83, .75, and .60 between self-reported health and activity limitations, activity limitations and poor health score, and self-reported health and poor health score respectively. The corresponding coefficients for men were somewhat lower than for women (.77, .64 and .44 respectively).

Women reported poorer health than men on all three measures. For the full data set, the excess fraction was 15 % for the health score based on the eight domains, 28 % for “poor” or “very poor” self-rated health on the single question, and 26 % for “severe” or “extreme” on the single question on limitations. The excess female reporting of poorer health occurs at all ages, but is smaller at ages 60 and over than below 60 years for all three measures. The gap in health score declines gradually with age and was smallest at 80 years and over. The gender gap based on the single self-rated health and on the activity limitations indicators peaked at ages 40–49 years.

The European country groups and sub-Saharan Africa had the smallest gender differences (Table 1). The largest female excess fractions were observed for the countries in the Latin America and South Asia regions as well as in the group “Other”.

**Fig. 1:**
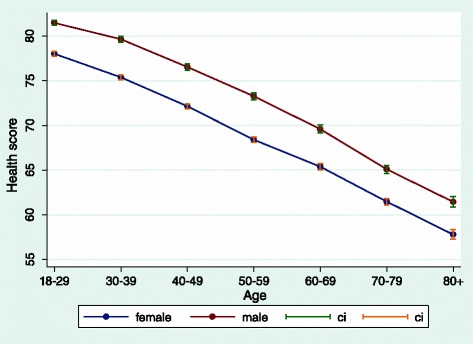
Levels of health pooled across the 59 surveys for men and women by age – mean levels and standard errors

The differences in the domain-based health score are observed in all 59 country surveys with the exception of Norway where women had a higher score than men. Women also reported poorer health on the single self-rated health question for 52 of 59 country surveys, with the exception of seven European countries (Estonia, Finland, Hungary, Latvia, Norway and Ireland), and for 46 of 51 countries on the limitations in daily activities question (the exceptions were Hungary, Latvia, Comoros, Ghana and Namibia). Figure 1 shows the pooled results of the differences between men and women in the health score across ages. Women are consistently in worse health than men across all age groups. This difference in the score is of about a decade of ageing – in other words a woman in a younger age group has the same level of health as a man in the next decade of age.

### Domain differences

The upper panel of Table 2 shows the relative female excess of “severe” or “extreme” reporting for each of the eight domains of the health score, each based on two questions. (We also examined excess reporting for the 16 individual questions of the self-reported health score. The emerging gender patterns were similar to those presented here.) The large differences between women and men occurred in all regions and for all eight domains. Overall, the remarkably small range of the excess fraction was 25 % for vision to 35 % for mobility, pain and sleep.

**Table 2:** Excess fraction women over men by health domains and algorithm-based diagnoses, by region, World Health Survey 2002–04

	Prevalence (%)					Excess (%)^a^			
	Women	Men	Total	Sub-Saharan Africa	Latin America	Europe, high income	Eastern Europe	South Asia	Other
Domains									
Mobility	23.7	15.4	35	35	44	19	26	35	41
Self-care	3.9	2.7	31	24	12	17	30	30	53
Pain	14.6	9.5	35	27	43	35	31	41	45
Cognition	10.5	7.0	33	27	45	14	22	36	46
Relationships	5.5	3.9	28	20	36	2	27	32	41
Vision	8.6	6.4	25	19	27	49	28	23	41
Sleep	12.5	8.1	35	26	46	49	31	41	39
Affect	15.2	11.1	27	26	43	59	43	13	19
Diseases based on algorithms									
Angina	12.9	8.7	33	36	42	15	37	26	33
Arthritis	9.2	6.3	32	21	42	24	35	33	36
Asthma	7.4	6.8	8	18	25	0	1	1	0
Depression	8.2	4.8	42	32	54	55	55	34	39
Diabetes^a^	3.8	3.3	14	16	30	−12	10	1	21

^a^Excess is the female excess fraction; for domains of health a score combining the two questions in each domain (see Additional file 1) was used; all female-male differences are statistically significant at the 1 % level; all figures are weighted and age-standardized

Regional patterns varied. In the high-income country group there was most variability, with large female excess for the domains of affect, sleep and vision and small for personal care, cognition and self-care. Other regions generally had much smaller variation in the gender gap by domain.

### Chronic conditions

The lower panel of Table 2 shows the excess fraction of a presumptive diagnosis by region. Angina, arthritis and depression had female excess fractions of 33, 32 and 42 % respectively, occurring in all regional groups. Depression had the largest excess fractions, especially in the European country groups and Latin America. For asthmatic conditions and diabetes the gender gaps were small overall and consistently so in all regions.

### Other differences

Overall, 19.9 % of women and 16.7 % of men were not satisfied with their own health, an excess fraction of 16 %. (detail in Additional file 4) The proportion rose with age for both sexes until ages 50–59, but the sex differences remained fairly constant from age 60.

Women were more frequent users of ambulant health services, but the differences were modest: 50 and 43 % of women and men respectively made at least one visit for ambulant health care in the last 12 months. Women had also been admitted more frequently to a hospital in the last 5 years: 25 and 17 % for women and men respectively. The greater use of inpatient services by women however was limited to ages 18–39 years, and presumably associated with the female reproductive ages, as the gender gap was negligible for ages 40 and over (see Additional file 4).

### Factors affecting the gender gap

Table 3 presents the results of the analysis of factors influencing the size of the country gender gaps for each of the three measures of self-reported health. The table only includes variables that were significantly associated with self-reported health measures at the 10 % level in a bivariate regression. No association was observed for many variables, including gender gaps in life expectancy, smoking and alcohol use. Region was not a significant factor with the exception of a smaller gap in self-rated health in high-income countries compared to all other countries which disappeared in the multivariate analysis.

**Table 3:** Association between the gender gap in three self-reported health measures and determinants, World Health Survey 59 countries (coefficients with *p*-value of *t*-test shown)

	Bivariate	Multivariate
A Domain poor-health score		
Employment gap	.0010 (.090)	.0174 (.012)
Education gap	.0132 (.100)	a
Life expectancy gap	−.0066 (0.09)	a
Depression gap	.0128 (<.001)	.0115 (<.001)
Arthritis gap	.0115 (.006)	.0084 (.033)
B Overall Self-rated health		
Gender inequality index	.0581 (.020)	a
High income countries	−.3350 (.027)	a
Islamic country	.1823 (.098)	a
Employment gap	.0059 (.007)	.0058 (.008)
Depression gap	.0310 (.026)	a
Arthritis gap	.0604 (.001)	.0051 (.008)
C Daily Activity limitations		
Depression gap	.0858 (.003)	a
Arthritis gap	.1180 (.001)	.0384 (.015)
Angina gap	.1010 (.001)	a
Diabetes gap	−.1575 (.013)	a

Bivariate regression column only includes variables with an association significant at the 10 % level; Multi-variate regression model only includes variables remaining significant at the 10 % level in a stepwise regression; (a) means removed from model because *p* > .10; Model A: *r* ^2^ = 0.37; model B: *r* ^2^ = 0.30; model C: : *r* ^2^ = 0.12

Greater gender inequality, as measured by the gender equality index and by gaps in employment and education were significant factors affecting the size of the females excess fraction in several models. The size of the gender gap was associated with the excess fraction for arthritis for all three measures of self-reported health. The depression prevalence gap between women and men was also associated with all three measures in bivariate analysis, but only in multivariate analysis only significantly so for the domain-based health score.

**Fig. 2:**
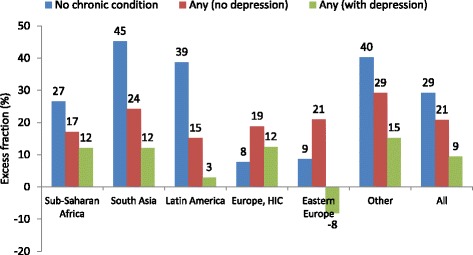
Women excess fraction for three health measures by different combinations of presumptive diagnosis of chronic conditions

Figure 2 shows the female excess fraction in self-reported health if there was no chronic condition; any chronic condition; but no depression, and any chronic condition including depression. The gender gap was considerably smaller if women and men had the same chronic conditions, especially if depression was also diagnosed. The patterns were similar in all regions, except the two European country groups.

## Discussion

The analysis of World Health Survey data from 59 countries shows a large and consistent gender gap in reporting of health problems in all regions. Women had much higher prevalence of poor health than men for each of three indicators of self-reported health, as well as eight functional domains. The overall consistency in the size of the gap among the eight domains was notable. In addition, women of all ages had higher prevalence of three algorithm-based diagnoses of depression, angina and arthritis. In general, regional patterns in the self-reported health gap between the sexes were also remarkably similar.

The size of the gender gap in self-reported health was influenced by both societal and biological factors. Gender gaps in employment and education, as well as greater gender inequality, were significant factors in some models. An individual-level analysis of self-reported health by gender of the World Health Survey also showed that some of the differences between women and men could be attributed to social determinants, mostly employment levels [8]. Behavioural factors such as smoking or alcohol differences between the sexes had no effect. Overall, the effects of gender inequality in societies, either measured through the gender inequality index, education or employment, appeared to be modest in terms of explaining why women report much poorer health than men. The level of fertility or mortality in a country did not explain the gender gap in self-reported health.

The consistency of the findings of across regions suggests that biological differences between women and men are a key factor contributing to the gender gaps in self-reported health. Our results from the composite domain health score show the dramatic difference between the levels of health between women and men – equivalent to a decade of ageing. The gender gap in self-reported health was associated with differences in the prevalence of chronic conditions, based on recall and symptom questions, notably arthritis and depression. Data from surveys in high-income countries have also suggested a substantive role of disease prevalence, as female-male differences were much smaller when the prevalence of self-reported chronic diseases and functioning were taken into account [2, 3]. There was also a larger gender gap between ages 40–59 years, perhaps related to the menopause or greater male underreporting but the study could not provide any insights into possible causes for this age pattern.

The higher prevalence of conditions among women can be due to higher incidence or lower case fatality rates. Women at all ages reported more chronic conditions that are non-fatal but are debilitating such as arthritis, depression, cognitive loss, asthma, but men may have more cardiovascular diseases and maybe more likely to suffer from acute conditions with higher case fatality [3, 30]. There was however no evidence of a survivor bias in the self-reported health, as the gender gaps did not increase with age. The opposite occurred in our analysis, which has also been observed in several studies in high-income countries [2, 31].

Depression as measured in the World Health Surveys was common, especially among women, and has been linked with higher self-reported levels of health problems [7]. The presumptive diagnosis was based on the full syndromal criteria for depression as described in the International Classification of Diseases [32] which is distinct from occasional mood fluctuations and correlates with well biological mechanisms [33]. In addition, previous analyses have shown that the decrements in health associated with depression are not attributable to reporting biases or the domains used in our analyses [8]. Depression may be an underlying factor for the gender gaps in self-reported health that affects all indicators. It is noted that the domain-based health score itself includes several mental health related items such as cognition, sleep and interpersonal relationships, which implies that the results have to be interpreted with caution. However, earlier analysis of the World Health Surveys showed that this is unlikely due to a bias resulting from the inclusion of affect related items in the overall poor-health score [7].

Health service utilization by women tends to be at least equal or higher than that for men in virtually all countries. Therefore, differential access and utilization of health services is not a likely reason for gender differences in the prevalence of health problems, unless the quality of treatment received differs greatly and in all societies. The more frequent contact with health services among women could contribute to the gender gap in reporting, due to higher awareness and reporting of recalled doctor’s diagnoses and symptoms.

In addition to the limitations discussed above it is noted that the quality of the World Health Surveys data is likely to be variable, as the capacity of the implementing agencies is likely to have varied between countries. The individual response rates were not significantly different between males and females in any country [34]. In spite of possible quality differences in country surveys the consistency of the gender gap in self-reported health is remarkable and gives confidence in the conclusion about its generalizability. Also the World Health Surveys did not include biological or clinical data which may throw further light on the gender differences. The WHO longitudinal study on adult health and ageing (SAGE), implemented in six countries, does include biological and clinical data collection and will be used for further analyses of self-reported health and gender gaps [35].

Single questions on overall self-rated health are commonly used to compute a population health summary measure, such as healthy life expectancy [4]. A substantial large drawback of such single overall self-reported health measures is the large counterintuitive variation between populations and over time [36]. One way to address this problem is to include a composite measure of self-reported health derived from difficulties in functioning in multiple domains as was done in our ‘poor-health’ measure.

The analysis focused on the gap in health across countries with different social, economic and cultural contexts and hence we included the country as a dummy variable in our analyses in addition to the other variables that could be construed as ‘ecological’. If health gap between the sexes is described as an individual level variable, multi-level modeling where individuals and country are treated as separate levels or include interaction terms between country and gender can be used to further disentangle gender differences in the different societal contexts. This could build upon a decomposition analysis that was conducted using the same data set to understand the determinants of health among men and women at the individual level [9].

## Conclusion

The consistent widespread gap in self-reported health measures between men and women observed in this study of 59 countries across the globe is remarkable and the gap should be taken into account in all work in search of summary measures of population health that include self-reported data and to examine increasing divergence between life expectancy and healthy life expectancy as populations age worldwide. The multi-country study suggests that a combination of societal and biological factors is contributing to the large differences between in self-reported health by women and men in all regions of the world.

## Additional files

Additional file 1: Country surveys. (DOCX 14 kb) Additional file 2: Questionnaire. (DOCX 23 kb) Additional file 3: Country data. (XLSX 20 kb) Additional file 4: Service utilization. (XLSX 10 kb)
