# Supplementary material for: A global assessment of the gender gap in self-reported health with survey data from 59 countries
Source: BMC Public Health. 2016 Jul 30;16:675. doi: 10.1186/s12889-016-3352-y (PMC4967305; doi:10.1186/s12889-016-3352-y)
Supplement: Additional file 1: — Country surveys. (DOCX 14 kb) [file 12889_2016_3352_MOESM1_ESM.docx]

Additional Table 1: Countries with surveys in the different groups in the study.

| Group | Countries |
| --- | --- |
| Europe, high income (10) | Czech Republic, Finland, France, Ireland, Luxemburg, Norway, Portugal, Spain, Sweden; Israel |
| Eastern Europe & Central Asia, middle income (11) | Bosnia & Herzegovina, Croatia, Georgia, Estonia, Hungary, Kazakhstan, Latvia, Russia, Slovakia, Turkey, Ukraine |
| Sub-Saharan Africa (18) | Burkina Faso, Chad, Comoros, Congo, Cote d'Ivoire, Ethiopia, Ghana, Kenya, Malawi, Mali, Mauritania, Mauritius, Namibia, Senegal, South Africa, Swaziland, Zambia, Zimbabwe |
| Latin America (6) | Brazil, Dominican Republic, Ecuador, Mexico, Paraguay, Uruguay |
| South Asia (6) | Bangladesh, India, Myanmar, Nepal, Pakistan, Sri Lanka |
| Eastern Asia (5) | China, Lao, Malaysia, Philippines, Vietnam |
| North Africa & Middle East (3) | United Arab Emirates, Morocco, Tunisia |
|  |  |
| Islamic (13) | Turkey; Burkina Faso, Chad, Comoros, Mali, Mauritania, Senegal; Bangladesh, Pakistan; Malaysia; UAE, Morocco, Tunisia |
|  |  |
