# Supplementary material for: A global assessment of the gender gap in self-reported health with survey data from 59 countries
Source: BMC Public Health. 2016 Jul 30;16:675. doi: 10.1186/s12889-016-3352-y (PMC4967305; doi:10.1186/s12889-016-3352-y)
Supplement: Additional file 2: — Questionnaire. (DOCX 23 kb) [file 12889_2016_3352_MOESM2_ESM.docx]

Additional file 2. Health module, World Health Survey.

|  | | **Time Begin: __ __ : __ __** | | | | | | | | | |  |
| --- | --- | --- | --- | --- | --- | --- | --- | --- | --- | --- | --- | --- |
|  | | **Overall Health** | | | | | | | | | |  |
|  | | The first questions are about your overall health, including both your physical and your mental health. | | | | | | | |  | | |
| Q2000 | | In general, how would you rate your health today? | | | VERY GOOD 1  GOOD 2  MODERATE 3  BAD 4  VERY BAD 5 | | | | |  | | |
| Q2001 | | Overall in the last 30 days, how much difficulty did you have with work or household activities? | | | NONE 1  MILD 2  MODERATE 3  SEVERE 4  EXTREME/CANNOT DO 5 | | | | |  | | |
|  | | Now I would like to review the different functions of your body. When answering these questions, I would like you to think about the last 30 days, taking both good and bad days into account. When I ask about difficulty, I would like you to consider how much difficulty you have had, on an average, in the past 30 days, while doing the activity in the way that you usually do it. By difficulty I mean requiring increased effort, discomfort or pain, slowness or changes in the way you do the activity. Please answer this question taking into account any assistance you have available. | | | | | | | |  | | |
|  | | [Read and show scale to respondent]. | | | | | | | |  | | |
|  | | **Mobility** | | | | | | | |  | | |
| Q2002 | | | Overall in the last 30 days, how much difficulty did you have with moving around? | | NONE 1  MILD 2  MODERATE 3  SEVERE 4  EXTREME/CANNOT DO 5 | | | | |  | | |
| Q2003 | | | In the last 30 days, how much difficulty did you have in vigorous activities (such as [country to define] WHS02=running 3 km (or equivalent) or cycling)? | | NONE 1  MILD 2  MODERATE 3  SEVERE 4  EXTREME/CANNOT DO 5 | | | | |  | | |
|  | | **Self-Care** | | | | | | | |  | | |
| Q2004 | | Overall in the last 30 days, how much difficulty did you have with self-care, such as washing or dressing yourself? | | | NONE 1  MILD 2  MODERATE 3  SEVERE 4  EXTREME/CANNOT DO 5 | | | | |  | | |
| Q2005 | | In the last 30 days, how much difficulty did you have in taking care of and maintaining your general appearance (e.g. grooming, looking neat and tidy etc.) | | | NONE 1  MILD 2  MODERATE 3  SEVERE 4  EXTREME/CANNOT DO 5 | | | | |  | | |
|  | | **Pain and Discomfort** | | | | | | | |  | | |
| Q2006 | | Overall in the last 30 days, how much of bodily aches or pains did you have? | | | NONE 1  MILD 2  MODERATE 3  SEVERE 4  EXTREME 5 | | | | |  | | |
| Q2007 | | In the last 30 days, how much bodily discomfort did you have? | | | NONE 1  MILD 2  MODERATE 3  SEVERE 4  EXTREME 5 | | | | |  | | |
|  | | **Cognition** | | | | | | | |  | | |
| Q2008 | | | Overall in the last 30 days, how much difficulty did you have with concentrating or remembering things? | | | | NONE 1  MILD 2  MODERATE 3  SEVERE 4  EXTREME/CANNOT DO 5  DON'T KNOW 8 | | |  | | |
| Q2009 | | | In the last 30 days, how much difficulty did you have in learning a new task (for example, learning how to get to a new place, learning a new game, learning a new recipe)? | | | | NONE 1  MILD 2  MODERATE 3  SEVERE 4  EXTREME/CANNOT DO 5 | | |  | | |
|  | | **Interpersonal Activities** | | | | | | | |  | | |
| Q2010 | | | Overall in the last 30 days, how much difficulty did you have with personal relationships or participation in the community? | | | | NONE 1  MILD 2  MODERATE 3  SEVERE 4  EXTREME/CANNOT DO 5 | | |  | | |
| Q2011 | | | In the last 30 days, how much difficulty did you have in dealing with conflicts and tensions with others? | | | | NONE 1  MILD 2  MODERATE 3  SEVERE 4  EXTREME/CANNOT DO 5 | | |  | | |
|  | | **Breathing** | | | | | | | | |  | |
| Q0212 | | Overall in the last 30 days, how much of a problem did you have with breathing, such as shortness of breath at rest? | | | | | | NONE 1  MILD 2  MODERATE 3  SEVERE 4  EXTREME/CANNOT DO 5 | | |  | |
| Q0213 | | Shortness of breath with mild exercise, such as climbing uphill for 20 meters or stairs (such as 12 steps) | | | | | | NONE 1  MILD 2  MODERATE 3  SEVERE 4  EXTREME/CANNOT DO 5 | | |  | |
|  | | **Vision (respondent should answer as when wearing glasses/contact lenses if used)** | | | | | | | |  | | |
| Q2014 | | When was the last time you had your eyes examined by a medical professional? | | | WITHIN THE LAST 12 MONTHS 1  1-2 YEARS AGO 2  3-4 YEARS AGO 3  5 OR MORE YEARS AGO 4  NEVER 5 | | | |  | | | |
| Q2015 | | Do you use eyeglasses or contact lenses to see far away (for example across the street)? | | | YES 1  NO 2 | | | |  | | | |
| Q2016 | | Do you use eyeglasses or contact lenses to see up close (for example at arms length, like when you are reading)? | | | YES 1  NO 2 | | | |  | | | |
| Q2017 | | In the last 30 days, how much difficulty did you have in seeing and recognizing a person or object you know across the road (from a distance of about 20 meters)? | | | NONE 1  MILD 2  MODERATE 3  SEVERE 4  EXTREME/CANNOT DO 5 | | | |  | | | |
| Q2018 | | In the last 30 days, how much difficulty did you have in seeing and recognizing an object at arm's length (for example reading)? | | | NONE 1  MILD 2  MODERATE 3  SEVERE 4  EXTREME/CANNOT DO 5 | | | |  | | | |
| Q2019 | | In the last 5 years, were you diagnosed with a cataract in one or both of your eyes (that is, an opacity in the lens of the eye)? | | | YES 1  NO 2  DON'T KNOW 8 | | | | 🡺2021  🡺2021 | | | |
| Q2020 | | In the last 5 years, have you had eye surgery to remove this cataract(s)? | | | YES 1  NO 2 | | | | |  | | |
|  | | **Hearing (respondent should answer as when wearing hearing aid if one is used)** | | | | | | | |  | | |
| Q2021 | | In general, how is your hearing without using a hearing aid? Would you say it is…? | | | | VERY GOOD 1  GOOD 2  MODERATE 3  BAD 4  VERY BAD 5 | | | | |  | |
| Q2022 | | Do you wear a hearing aid | | | | YES 1  NO 2 | | | | |  | |
| Q2023 | | In the last 30 days, how much difficulty did you have in: hearing someone talking on the other side of the room in a normal voice (even with your hearing aid on if you use one)? | | | | NONE 1  MILD 2  MODERATE 3  SEVERE 4  EXTREME/CANNOT DO 5 | | | | |  | |
| Q2024 | | In the last 30 days, how much difficulty did you have in: hearing what is said in a conversation with one other person in a quiet room (even with your hearing aid on if you use one)? | | | | NONE 1  MILD 2  MODERATE 3  SEVERE 4  EXTREME/CANNOT DO 5 | | | | |  | |
|  | | **Sleep and Energy** | | | | | | | | |  | |
| Q2025 | | Overall in the last 30 days, how much of a problem did you have with sleeping, such as falling asleep, waking up frequently during the night or waking up too early in the morning? | | | | NONE 1  MILD 2  MODERATE 3  SEVERE 4  EXTREME 5 | | | | |  | |
| Q2026 | | In the last 30 days, how much of a problem did you have due to not feeling rested and refreshed during the day (e.g. feeling tired, not having energy)? | | | | NONE 1  MILD 2  MODERATE 3  SEVERE 4  EXTREME 5 | | | | |  | |
|  | | **Affect** | | | | | | | | |  | |
| Q2027 | | Overall in the last 30 days, how much of a problem did you have with feeling sad, low or depressed? | | | | | NONE 1  MILD 2  MODERATE 3  SEVERE 4  EXTREME 5 | | | |  | |
| Q2028 | | Overall in the last 30 days, how much of a problem did you have with worry or anxiety? | | | | | NONE 1  MILD 2  MODERATE 3  SEVERE 4  EXTREME/CANNOT DO 5 | | | |  | |
|  | | **Bowels and bladder** | | | | | | | | |  | |
| Q2029 | | In the last 30 days, how much difficulty have you had with urinating or controlling your urine? | | | | | NONE 1  MILD 2  MODERATE 3  SEVERE 4  EXTREME/CANNOT DO 5 | | | | 🡺 2112 | |
| Q2030 | | On about how many days in the last month has this happened to you? | | | | | <5 DAYS 1  5-14 DAYS 2  >15 DAYS 3  DK 8 | | | |  | |
| Q2031 | | In the last 30 days, how much difficulty have you had with defecating, including constipation? | | | | | NONE 1  MILD 2  MODERATE 3  SEVERE 4  EXTREME/CANNOT DO 5 | | | | 🡺 2120 | |
| Q2032 | | On about how many days in the last month has this happened to you? | | | | | <5 1  5-14 DAYS 2  >15 DAYS 3  DK 8 | | | |  | |
|  | | **Digestion** | | | | | | | | |  | |
| Q2033 | | In the last 30 days, for how many days did you have a problem due to: burning in the stomach? | | | | | | <5 1  5-14 DAYS 2  >15 DAYS 3  DK 4 | | |  | |
| Q2034 | | In the last 30 days, for how many days did you have a problem due to: loose stools 3 or more times a day? | | | | | | <5 1  5-14 DAYS 2  >15 DAYS 3  DK 4 | | |  | |
|  | | **Skin and disfigurement:** Now I would like to ask you some questions about your physical appearance. | | | | | | | | |  | |
| Q2035 | | Have you had a problem with a skin defect of face, body, arms or legs? | | | | | | NONE 1  MILD 2  MODERATE 3  SEVERE 4  EXTREME 5 | | | 🡺 2200 | |
| Q2036 | | Have you had a problem with your appearance due to missing or deformed or paralyzed arms, legs, feet? | | | | | | NONE 1  MILD 2  MODERATE 3  SEVERE 4  EXTREME 5 | | |  | |
